# Modulation of the Immune System in Chronic Hepatitis C and During Antiviral Interferon-Free Therapy

**DOI:** 10.1007/s00005-018-0532-8

**Published:** 2018-11-15

**Authors:** Arkadiusz Urbanowicz, Radosław Zagożdżon, Michał Ciszek

**Affiliations:** 1 Department of Immunology, Transplant Medicine and Internal Diseases, Medical University of Warsaw, Warsaw, Poland; 2 Department of Clinical Immunology, Medical University of Warsaw, Warsaw, Poland; 3 Institute of Biochemistry and Biophysics, Polish Academy of Sciences, Warsaw, Poland

**Keywords:** HCV, Immune system, Direct-acting antivirals, Hepatocellular carcinoma

## Abstract

The treatment of patients with chronic hepatitis C virus (HCV) infection has changed tremendously over the past 2 years, with an increasing variety of all-oral direct-acting antiviral (DAA) treatment regimens available for different HCV genotypes and distinct clinical settings. These treatments have significantly improved safety in patients with advanced liver disease compared with interferon (IFN)-based regimens. HCV modifies the human immune system to escape immunosurveillance via several mechanisms. One of the basic mechanisms of HCV is the ability to “switch” the immune response by reducing the activity of cells responsible for the elimination of virus-infected cells. IFN-free DAA treatment regimens provide a unique opportunity to assess the effect of HCV elimination on the immune system. Abrupt changes in the immune system can in some cases be responsible for two alarming processes: viral reactivation in patients with chronic hepatitis B and recurrence of hepatocellular carcinoma in patients with previous successful cancer treatment.

## Introduction

Hepatitis C virus (HCV) affects the immune system in multiple ways, allowing HCV to evade recognition and effective elimination by the host. Specific changes in the innate and adaptive immune responses that promote persistence of infection are observed in patients with chronic hepatitis C. Interferon (IFN)-α-based treatments rely on enhancing the native antiviral mechanisms of the host immune system. However, the effect of inhibition of viral replication on the immune system has been difficult to assess. The introduction of direct-acting antiviral drugs (DAAs) offers a unique opportunity to evaluate the changes in the immune system of the host caused solely by inhibition of viral replication. At present, three different classes of DAAs are available, and they differ based on the specific viral protein they target: NS3/4A protease inhibitors (simeprevir, paritaprevir, grazoprevir, voxilaprevir and glecaprevir), NS5A inhibitors (daclatasvir, ledipasvir, ombitasvir, elbasvir, velpatasvir, and pibrentasvir) and NS5B polymerase inhibitors, which block replication of viral RNA (sofosbuvir and dasabuvir). Achievement of a sustained virologic response (SVR) is the commonly accepted measure of DAA treatment efficacy. It has not been established to date if any other criteria, such as immunologic, could be helpful in evaluating the balance between efficacy and the risk of adverse effects in patients treated with DAA. At present, we can evaluate the effects of DAAs only in the short term, as longer-term follow-up is still ongoing in the treated population (Jakobsen et al. 2017). However, abrupt changes in the immune system may in some cases be responsible for two alarming developments: hepatitis B reactivation (Aggeletopoulou et al. 2017) and increased risk of hepatocellular carcinoma (HCC) recurrence in patients with previous successful treatment of HCC (Debes et al. 2017).

This article summarizes the interactions between HCV and the immune system in patients with chronic hepatitis C and the way that DAAs affect this interplay. We also discuss the mechanisms of the possible risk of an increase in HCC recurrence via inhibition of HCV replication.

## Immune System Responses to Chronic Hepatitis C

### Innate Immune Response

The innate immune response is the first line of defense in acute HCV infection. Different types of IFN, mostly class I (IFN-α and -β), are the essential factors that stimulate an antiviral response. IFNs activate the expression of a number of IFN-stimulated genes (ISG), leading to restriction and eradication of infection, although the level of gene expression is quite variable between individuals (Wieland et al. 2014). After HCV particle uptake into the hepatocyte, pattern recognition receptors such as RNA helicase retinoic acid-inducible gene-1 (RIG-1) and melanoma differentiation antigen 5 (MDA-5) bind viral RNA. Concurrently, viral particles and apoptotic bodies containing HCV RNA that have been phagocytosed by macrophages and dendritic cells (DCs) are degraded in endosomes, and the released viral RNA binds to Toll-like receptors (TLR) 3, 7 and 8. As a result, NF-κB transcription factor and interferon regulatory factor (IRF)-3 and/or -7 become activated, and the latter binds to the promoter regions of genes encoding IFN class I and III (Horner and Gale 2013). However, the details of the signaling cascades via TLR3, 7 and 8 vary. For instance, TLR3 primarily senses double-stranded RNA and then, via IRF-3, induces the production of type I IFN (Wang et al. 2009). TLR7 and 8, in turn, are sensors for single-stranded RNA and transduce the signal via myeloid differentiation primary-response protein (MyD88). However, TLR7 signaling tends to induce higher production of type I IFN, whereas activation of TLR8 induces more pronounced generation of pro-inflammatory cytokines (McGilvray et al. 2012). TLR-mediated recognition of HCV infection is considered a significant player in the host antiviral response. Indeed, defective production of TLR3- and TLR7/8-induced cytokines is suspected to be one of the causative factors in aggressive HCV recurrence after liver transplantation, mainly via deregulation of the immune response to HCV, which causes subsequent activation of fibrogenesis (Howell et al. 2013).

Among the TLR receptors mentioned above, signal transduction via TLR7 is crucial for the proper activation of DCs and T cell priming. In humans, TLR7 has been detected mainly on plasmacytoid DCs (pDCs) and occasionally also on myeloid DCs. Observations in animal models deficient in TLR signaling pathways have underscored the substantial role of TLR7 in antiviral responses. Importantly, some studies have reported impaired activation of T cells in response to the specific antigen in mice lacking MyD88, indicating disrupted TLR7 signaling. Accordingly, the potent antiviral activity of a number of TLR ligands has been further studied. For instance, application of the TLR7 agonist isatoribine has been shown to markedly enhance viral clearance by the immune system, as monitored by dose-dependent dynamics of immunological biomarkers (Horsmans et al. 2005). Furthermore, when ANA773, an oral isatoribine prodrug, was administered to HCV-infected patients every second day for 28 days (800, 1200 or 1600 mg) or for 10 days (2000 mg), a detectable decrease in serum HCV RNA was reported in the 2000 mg-dosed group (Fidock et al. 2011). Importantly, in addition to the nonspecific inflammatory responses, TLR activation can enhance antigen-specific immunity to viral proteins and may contribute to improved treatment of HCV strains resistant to DAAs, as well as the development of an effective anti-HCV vaccine (Du et al. 2018).

However, contradicting data on the role of TRL7 in HCV infection have also been reported. For instance, Mele et al. (2016) described a dose-dependent inhibition of cytokine production and expression of activation markers in CD4 T cells, which were rescued by a TLR7-specific antagonist. These findings may indicate that under certain circumstances, HCV induces CD4 T-cell dysfunction via TLR7-mediated signaling, which may then contribute to defective virus eradication. These observations indicate a need for caution in the administration of TLR7 agonists to enhance the immune response in chronic RNA virus infections. More detailed studies are necessary to clarify this issue.

IFN molecules released from infected cells bind to membrane receptors (IFN-α/β receptor, IFNAR) on adjacent hepatocytes and immune cells, resulting in activation of JAK kinases, which in turn phosphorylate signal transducer and activator of transcription (STAT). The phosphorylation of STAT leads to the activation of hundreds of IFN-dependent genes directly through STAT1 homodimers and STAT1/STAT2 heterodimers combined with IRF-9 and to mRNA transcription for a series of antiviral proteins (Sadler and Williams 2008). The degree of ISG activation varies in patients with chronic hepatitis C; enhanced expression of hundreds of genes induced by IFN I and III in the liver is observed only in approximately half of patients (Wieland et al. 2014). ISG activation, however, does not result in the elimination of HCV infection, and IFN-α therapy has been found to be less effective in these patients (Sarasin-Filipowicz et al. 2008).

Within the immune system, type I IFNs (comprising several IFN-α and one IFN-β) and type III IFNs (IFN-λ1, -λ2, and -λ3) are produced primarily by macrophages and DCs. Interestingly, DCs and macrophages constantly scour their surroundings for virions or cell remnants possibly containing viral particles. As a consequence, they can secrete IFNs even when are not infected by the virus. Plasmacytoid DCs in HCV-infected livers have been demonstrated to produce type I IFN via TLR7-mediated signaling without becoming infected (Narita et al. 2009). It is anticipated that some type of cellular communication must be involved in the process of endocytosis of HCV RNA particles from infected hepatocytes by pDCs. CD81/CD9 tetraspanins have been proposed as mediators of such cross-talk between cells (Zhang et al. 2013). A recent review article also suggested a role for extracellular vesicles in the regulation of TLR-mediated antiviral responses (Kouwaki et al. 2017).

NK cells are the key effector cells of the innate immune response. In the absence of infection or inflammation, NK cell activity is suppressed by the interaction of their inhibiting KIR (killer-cell immunoglobulin-like) receptors and NKG2A/CD94 with MHC particles located on noninfected cells. In HCV infection, NK cells exert a direct antiviral effect through (1) perforin and tumor necrosis factor-related apoptosis-inducing ligand (TRAIL)-dependent cytolysis of infected cells and (2) induction of the synthesis of type II IFN (IFN-γ). IFN-γ has multiple effects, i.e., stimulation of antigen presentation by macrophages, enhancement of Th1 differentiation and increased expression of MHC particles (Schroder et al. 2004). Through the abovementioned mechanisms, NK cells play a key role in immune cell recruitment to the infection site and the initiation of the adaptive immune response (Vivier et al. 2011). In chronic hepatitis, NK cells become activated, but their typical responses are disordered (Nattermann et al. 2006). This specific functional polarization of NK cells, which is characterized by increased cytolytic activity and decreased IFN-γ synthesis, is related to chronic exposure to IFN-α and may prevent the elimination of the virus, leading to chronic hepatitis. Increased expression of NK cell stimulation markers (CD69, HLA-DR) and activating receptors (NKp30, NKp44, NKp46, NKG2D, and CD122) is observed on NK cells (Rehermann 2015). This functional shift is probably a result of increased signaling via the natural cytotoxicity receptors NKp30, NKp44, and NKp46, which leads to the release of more perforin/granzyme-containing granules (Varchetta et al. 2012). Enhanced expression of the T-cell inhibitory receptor Tim-3 (T-cell immunoglobulin- and mucin-domain-containing molecule-3) has also been observed in NK cells, accompanied by increased cytolytic activity (Golden-Mason et al. 2015). Chronic exposure to endogenous IFN-α leads to increased expression of STAT1 in NK cells (Miyagi et al. 2010), which increases cytolytic activity (as measured by the degree of degranulation and expression of TRAIL), with simultaneous decreases in IFN-γ and tumor necrosis factor (TNF)-α synthesis (Ahlenstiel et al. 2010; Oliviero et al. 2009).

### Adaptive Immune Response

A delay in the development of the adaptive immune response is typical for HCV infection and may extend for up to 8–12 weeks after infection. In rare cases of spontaneous HCV elimination (up to 20–30%), the emergence of numerous specific CD4^+^ T cells and higher levels of interleukin (IL)-2, IFN-γ, TNF-α, and IL-17A are observed compared to patients with chronic hepatitis C (Missale et al. 1996; Park and Rehermann 2014). In most patients who have spontaneously eliminated HCV, a strong polyclonal response of CD4^+^ T cells is detected for a long time after clearance of the virus, in contrast to the chronic hepatitis scenario, where this response frequently fails to appear (Chang et al. 2001; Fernández-Ponce et al. 2017). Furthermore, an effective Th1/Th17 response, the proliferation of IL-21-producing cells and an increase in IL-21 blood levels are also observed exclusively in cases of spontaneous virus elimination. In chronic hepatitis C, a decrease in the T helper (Th)17 lymphocyte subpopulation producing IL-2 appears to be chiefly responsible for the lack of activation of virus-specific CD8^+^ lymphocytes (Kared et al. 2013). Patients failing to eliminate the virus had increased expression of proliferation-inhibiting receptors in CD4^+^ T cells, i.e., TIM-3, programmed death (PD)-1, cytotoxic T-cell antigen (CTLA)-4, and increased regulatory T lymphocytes (Treg) counts (Raziorrouh et al. 2011).

In the early phase of HCV infection, specific CD8^+^ lymphocytes show low proliferative potential and IFN-γ production but enhanced expression of PD-1, which stimulates their apoptosis (Kasprowicz et al. 2008). In cases of spontaneous elimination of the virus in the acute phase of HCV infection, decreased expression of PD-1 and increased expression of the anti-apoptotic Bcl-2 protein on CD8^+^ lymphocytes are observed (Urbani et al. 2006). In the course of chronic hepatitis C, the number of HCV-specific T lymphocytes found in the liver increases. However, their proliferative potential and IFN production are deficient (Klenerman and Thimme 2012; Neumann-Haefelin and Thimme 2013; Rehermann 2009). Impaired function of CD8^+^ lymphocytes is accompanied by increased expression of inhibiting receptors, such as PD-1, and by weak expression of the receptor for IL-7 (CD127) (Radziewicz et al. 2007, 2008). Antibodies that block PD-1 can partially reverse the state of specific suppression of HCV-specific CD8^+^ lymphocytes (Golden-Mason et al. 2007; Penna et al. 2007). However, inhibition of PD-1 is not sufficient to completely reverse the stunned phenotype of HCV-specific CD8^+^ lymphocytes in the liver, and inhibition of other receptors, such as CTLA-4 or TIM-3, might be necessary to restore T-cell functionality (McMahan et al. 2010; Nakamoto et al. 2008, 2009). Enhanced expression of TIM-3 appears to be a distinctive feature of suppressed HCV-specific CD8^+^ lymphocytes in the liver (Kroy et al. 2014). Impairment of the HCV-specific response in CD8^+^ lymphocytes is also a result of increased expression of a number of inhibiting receptors, such as 2B4, KLRG1 and CD160 (Bengsch et al. 2010; Schlaphoff et al. 2011). Regulatory CD4^+^CD25^+^ T cells can inhibit the function of CD8^+^ lymphocytes via direct intercellular interactions and by an increase in IL-10 production (Franceschini et al. 2009). IL-10-neutralizing antibodies can block the function of these regulatory lymphocytes (Abel et al. 2006; Accapezzato et al. 2004). In summary, the increased expression of inhibiting receptors, the lack of function of CD4^+^ cells, and the increased activity of Tregs all contribute to the impaired function of CD8^+^ lymphocytes in chronic hepatitis C (Boettler et al. 2005; Cabrera et al. 2004; Rushbrook et al. 2005; Semmo et al. 2005).

A few authors have also suggested that an imbalance between the Th1 and Th2 responses may be involved in the development of chronic hepatitis C. Changes in IL-4 and IL-13 serum and liver tissue levels may indicate a depressed Th1 and overactive Th2 response (Prezzi et al. 2001). HCV is exceptionally capable of infecting not only hepatocytes but also lymphocytes, as well as possibly other cells and tissues (Kondo and Shimosegawa 2013). This broad spectrum is attributable to the presence of the same receptor on hepatocytes and lymphocytes, i.e., tetraspanin (CD81). Tetraspanins comprise a large family of transmembrane proteins with the ability to costimulate T cells in vitro. Tetraspanin (CD81, CD82 or CD9) binding to CD28 in vitro leads to proliferation of naïve T cells. As a result of activation via this pathway, naïve T cells (both CD4^+^ and CD8^+^) differentiate into effector cells of the Th2 response and produce IL-4, IL-5, IL-13, and IL-10 along with IL-2 and TNF-α, but they do not synthesize IFN. The HCV E2 envelope protein is the only one known ligand for CD81. Therefore, it is postulated that activation of T cells independent of antigen presentation can occur in HCV-infected patients and contribute to the development of chronic hepatitis C via inhibition of the type 1 response and periodic overactivation of the type 2 response (Serra et al. 2008). Naive CD8^+^ and CD4^+^ T cells activated by binding of CD28 and CD81 differentiate into memory and effector cells. These cells are unable to produce IFN-α but are capable of producing considerable amounts of cytokines specific for the Th2-type response, such as IL-4, IL-13, IL-5, and IL-10; the latter additionally inhibits the Th1-type response (Messi et al. 2003). Interaction of c protein with the gC1q receptor located on the surface of monocytes/macrophages results in decreased production of IL-12, a cytokine that activates the Th1 response via increased production of IFN-γ (Eisen-Vandervelde et al. 2004). In T cells incubated with c protein, the same mechanism is responsible for enhanced expression of PD-1 (Yao et al. 2007). Expression of c protein and NS3 in DCs impairs their ability to activate HCV-specific T cells, which is probably related to the apparent increase in IL-10 and decrease in IL-12 production by DCs and results in impaired proliferation of HCV-specific T cells (Sarobe et al. 2003). In contrast to T cells, c protein is capable of activating B cells, resulting in enhanced surface expression of the activation marker CD69. This further leads to increased proliferation of B cells, production of IgM and IgG antibodies, and expression of surface costimulation markers, i.e., CD86 (B7-2), CD154 (CD40L), and CD195 (CCR5). Overactivation of B cells contributes to the development of cryoglobulinemia and its consequences, as well as non-Hodgkin lymphoma in the course of chronic hepatitis C (Lauletta et al. 2012).

Germinal center B cell selection is mediated by follicular T helper (Tfh) cells after the former have completed proliferation and somatic hypermutation (Crotty 2014). During chronic infection, the immune system promotes humoral responses, which bear less immune-pathological risk compared to cytotoxic T-cell responses, via enhanced differentiation and maintenance of Tfh (CXCR5^+^CD4^+^) cells. Tfh cells contributes to tolerance of chronic infection and are crucial for the maturation and adaptation of the antibody response and the formation of virus-neutralizing antibodies, which may be capable of controlling chronic infection (Fahey et al. 2011). However, persistently high levels of Tfh cells may also lead to a process of less rigorous B cell selection in germinal center responses, which eventually may lead to the activation of nonvirus-specific B cells, including autoreactive B cells and hypergammaglobulinemia (Greczmiel and Oxenius 2018).

The results of studies of CXCR5^+^CD4^+^ T cells, which represent a circulating subset of Tfh cells, support the role of Tfh cells in the immune response to HCV infection (Morita et al. 2011). The rate of CXCR5^+^CD4^+^ T cells is markedly higher in chronic hepatitis C patients than in healthy controls. The frequencies of CD19^+^ B cells, CD19^+^CD27^+^ B cells, or CD19^+^CD38^+^ B cells are comparable in both groups. Chronic hepatitis C patients show higher expression of CD4^+^ Th lineage-associated cytokines, with the exception of IL-21. Therapy with PEG-IFN/ribavirin results in elevated CXCR5^+^CD4^+^ T cell counts and reduced PD-1^+^ CXCR5^+^CD4^+^ T cell counts in patients with rapid virological response (RVR) compared with non-RVR patients (Zhang et al. 2016).

Interestingly, DAA therapy has been reported to increase Treg counts and decrease Tfh cell counts in patients (Comarmond et al. 2017).

## Clinical Consequences of the Impact of HCV on the Immune System

Dysregulation of the immune system by chronic HCV infection can trigger a number of autoimmune background processes. Autoimmune hepatitis in patients with HCV infection is the most prominent example, but a cause-and-effect relationship has not been sufficiently proved (Rigopoulou et al. 2013). There are numerous reports on extrahepatic autoimmune conditions in individuals with chronic hepatitis C (Retamozo et al. 2017). Autoantibodies are detected more often in the sera of persons chronically infected with HCV (especially with genotype 1) than in other populations (Lapinski et al. 2016). This results in a higher incidence of Sjögren’s syndrome (Wang et al. 2014), rheumatoid arthritis or systemic lupus erythematosus (Cacoub and Comarmond 2017; Mahroum et al. 2017). HCV-infected patients are prone to cryoglobulinemic vasculitis, autoimmune thyroiditis, glomerulonephritis, thrombocytopenia and skin diseases (e.g., psoriasis, lichen planus) (Cacoub et al. 2015; Ferri et al. 2017; Sayiner et al. 2017; Shen et al. 2016). An autoimmune basis is also postulated in HCV-related pulmonary fibrosis or porphyria (Aliannejad and Ghanei 2011). Furthermore, some autoimmune processes appear or are exacerbated during IFN-α-based treatment as a result of the immune-stimulatory properties of IFN (Dhillon et al. 2010; Tsuchiya et al. 2017; Wang et al. 2017). DAA treatment is believed to be generally safer in individuals with chronic HCV infection and concomitant autoimmune disorders (Cacoub et al. 2017; Yoshikawa et al. 2017).

## Changes in the Immune System During Treatment with DAA

Abrupt inhibition of HCV replication during DAA therapy leads to reversal of the phenotypic and functional shift of NK cells typical of chronic hepatitis C (Mondelli 2015). The expression of NK cell activation markers, e.g., HLA-DR, NKp46, CD85j, and NKG2A, normalizes and reaches levels similar to those in uninfected controls after the HCV viral load becomes undetectable. Concurrently, the expression levels of CD107a, a marker of NK cell degranulation/cytotoxicity, decrease, and the percentage of the IFN-γ-producing NK cell subset increases in patients with undetectable viremia (Serti et al. 2015). In addition, the rate of NK cells presenting TIM-3, a marker protein for enhanced cytolytic activity, decreases after successful treatment with DAAs (daclatasvir, asunaprevir, and BMS-791325). Decreased expression of T-bet and enhanced expression of Eomes have been observed after therapy, indicating a decrease in the cytolytic activity of NK cells after elimination of HCV (Burchill et al. 2015). Another author reported a decrease in proinflammatory cytokine levels, i.e., IP-10, monocyte chemoattractant protein 1 (MCP-1), macrophage inflammatory protein-1β (MIP-1β), and IL-18, which were paralleled by a drop in viremia during DAA treatment (Carlin et al. 2015). These changes were accompanied by a decrease in NK cell activation in the liver and peripheral blood and a subsequent decrease in their cytolytic activity and recovery of their normal phenotype during 8 weeks of antiviral treatment. This effect persisted after the completion of treatment. The expression of activating receptors on the NK cell surface in peripheral blood, i.e., NKp30 and NKp46, and the inhibiting receptor NKG2A are also decreased, similar to the reduced expression of TRAIL, suggesting a TRAIL-dependent decrease in the cytolytic activity of NK cells (Spaan et al. 2016).

In most patients, successful 12-week DAA treatment results in recovery of the HCV-specific CD8^+^ T-cell response, restoration of the CD4^+^ T-cell compartment and replenishment of the effector memory T-cell population (Burchill et al. 2015; Martin et al. 2014). No increase in the CD8^+^ T-cell response specific for influenza virus or cytomegalovirus has been found, and hence the specific effect of HCV elimination appears to be responsible, rather than the immunomodulatory effect of antiviral drugs. In cell cultures of HCV-infected hepatocytes, CD8^+^ T cells harvested from patients who completed DAA treatment were highly active in terms of antiviral potential, as measured by the degree of proliferation and cytolytic activity (Martin et al. 2014). Reinstatement of cytotoxic T-cell activity was accompanied by decreased expression of PD-1 on the surface of HCV-specific CD8^+^ T cells (Burchill et al. 2015). Notably, recovery of the specific CD8^+^ response in chimpanzees after successful DAA treatment 2 years earlier did not prevent the development of chronic hepatitis C after HCV reinfection (Callendret et al. 2014).

## Clinical Consequences of Immune Reconstruction During Treatment with DAA

### Hepatitis B Reactivation

Hepatitis B virus (HBV) reactivation, defined as an abrupt increase in HBV replication in patients with inactive or resolved HBV infection, is a newly identified safety issue in patients with hepatitis C treated with DAAs and coinfected with HBV. In a recently published study, nine (eight HBsAg-positive and one isolated hepatitis B core antibody-positive) of 62,290 patients treated with DAAs had evidence of HBV reactivation occurring on therapy. Seventeen other patients had small increases in HBV DNA levels that did not qualify as HBV reactivation (Belperio et al. 2017). In another study, HBV reactivation occurred earlier and was clinically more significant in chronic hepatitis C patients coinfected with overt or occult HBV treated with DAAs compared with IFN-based therapy. Although the pooled incidence rate of HBV reactivation among hepatitis C patients with overt HBV was similar in both groups, it was reported to occur much earlier in those treated with DAAs than in those treated with IFN-based therapies. In addition, hepatitis due to HBV reactivation was more frequent in the case of DAA-based therapies. Virus reactivation also occurred, although less frequently, in hepatitis C patients with occult HBV infection treated with DAAs (Chen et al. 2017). Testing for evidence of HBV infection is recommended in all HCV-infected patients before initiating DAA treatment. Patients with evidence of current or prior HBV infection require clinical monitoring while receiving DAA therapy (Bersoff-Matcha et al. 2017).

### Hepatocellular Carcinoma Recurrence

Reduction of the cytolytic activity of NK cells may result in specific inhibition of cancer surveillance of the immune system. In a study of 101 chronic hepatitis patients treated with DAAs, 12 experienced a rapid recurrence of HCC after treatment completion. Enhanced expression of NKG2D in NK cells before treatment and a more abrupt reduction of NKG2D expression during treatment were related to the risk of HCC recurrence (AUC = 0.92). Decreased expression of NKG2D in NK cells was also inversely related to Treg (CD25^high^CD127^−^CD4^+^) count, which could reduce the expression of NKG2D through transforming growth factor-β secretion (among other mechanisms) (Chu et al. 2017). Decreased expression of the NKG2D activating receptor and an increased number of CD25^+^ T cells can adversely affect immune surveillance of concealed cancer cells and may be responsible for the rapid recurrence of HCC after DAA therapy (Ghiringhelli et al. 2006).

Interestingly, in patients infected with HBV or HCV, when HCC and cancer-free liver tissue of 87 patients with HCC were compared, TLR7 expression was significantly downregulated in neoplastic hepatocytes (*p* < 0.001). Moreover, in a hepatoma cell line (HepG2), IFN-γ was reported to markedly reduce TLR7 promoter activity and expression in a dose-dependent manner. Hepatitis virus may promote downregulation of TLR7 gene expression via IFN-γ to modulate inflammatory signaling in hepatoma cells (Chang et al. 2010; Lin et al. 2012).

The rate of circulating CXCR5^+^CD4^+^ T lymphocytes was significantly reduced in HCC patients compared to patients with hepatic cirrhosis harboring HBV infection and healthy controls. In addition, the reduction in circulating CXCR5^+^CD4^+^ T cells correlated with disease progression. The percentage of CXCR5^+^CD4^+^ infiltrated T cells was significantly reduced in tumor regions compared to invariant regions. In addition, compared to the healthy control group, the functional circulating CXCR5^+^CD4^+^ T-cell population in HCC was deteriorated and characterized by the diminished production of IL-21 and deregulation of the promotion of B cells. Notably, the control data indicated that the reduced frequency of circulating CXCR5^+^CD4^+^ T cells was also associated with a reduction of disease-free survival in patients with HCC (Jia et al. 2015).

An increased risk of cancer recurrence after treatment with DAAs was observed in some studies of patients with a history of HCC. It was first described in a study from Italy, where during 24 weeks of follow-up after treatment, HCC was detected in 26 patients: 17 of 59 patients (28.81%) with previous HCC and 9 of 285 patients (3.16%) without previous HCC. In multivariate analysis, Child-Pugh class (*p* = 0.03) and history of HCC (*p* < 0.0001) were independently associated with HCC recurrence (Conti et al. 2016). In another study including 58 patients with a prior history of treated HCC with complete remission, three patients died and 16 developed radiologic tumor recurrence (27.6%) at a median follow-up of 5.7 months after DAA treatment (Reig et al. 2016). Patients who developed HCC were younger; in 50%, HCC was multinodular, and in 20%, extrahepatic tumors were present. In a multicenter study from Spain with 70 patients with a prior diagnosis of HCC who had experienced a complete response before initiation of DAA therapy, HCC recurred in 21 (30%) within 12 months of starting DAA therapy, and two patients subsequently died. Comparatively, incident HCC was confirmed in 30/3233 (0.93%) patients without a prior diagnosis within 18 months of starting DAA therapy (Calleja et al. 2017). By contrast, in a French prospective, multicenter ANRS cohort that included more than 6000 patients treated with DAAs, no increased risk of cancer recurrence was observed in patients with HCC successfully treated before DAA therapy. In the HEPATHER cohort, 189 patients with chronic hepatitis C previously treated for HCC received DAA, and 78 did not; the rates of recurrence were 0.73/100 and 0.66/100 person-months, respectively. In the ANRS CO23 “Compassionate use of Protease Inhibitors in viral C Liver Transplantation” (CUPILT) cohort, 314 liver transplant recipients with a history of HCC were treated with DAAs. Seven HCC recurrences were reported after a median time of 70.3 months after liver transplantation; the rate of recurrence was 2.2% (ANRS collaborative study group 2016). In a retrospective cohort study of 22,500 patients treated with DAAs, there were 271 new cases of HCC, including 183 in patients with SVR. Compared to patients without SVR, those with SVR had a significantly reduced risk of HCC (adjusted hazard ratio: 0.28) (Kanwal et al. 2017).

Protease inhibitors reduce the incidence of cancer in HIV patients, and thus it is unlikely that DAAs could directly stimulate tumor cell growth. It was suggested that the reason for the increased recurrence rate of HCC is the rapid reduction in immune surveillance caused by a quick decrease in viremia (Nault and Colombo 2016). As a consequence, the European Medicine Agency (EMA) recommended including the risk of early liver cancer recurrence in further studies (EMA documents see at: http://www.ema.europa.eu/docs/en_GB/document_library/EPAR_-_Assessment_Report_-_Variation/human/003839/WC500223374.pdf).

It is important to remember that advanced fibrosis including cirrhosis is an independent risk factor for HCC, and this risk is still present after HCV eradication. Consequently, one should account for the risk of HCC in patients with advanced fibrosis and cirrhosis, even if they achieved SVR (van der Meer et al. 2017). In a study of HCV patients who achieved SVR with IFN treatment, the general yearly posttreatment incidence rate of HCC was reported to be 0.33%. Cirrhosis (1.39%) and age over 64 (0.95%) were found to be of particular risk for HCC in this group. Hence, life-long HCC surveillance in patients with cirrhosis should be considered, regardless of SVR status (El-Serag et al. 2016). Most recent reports are reassuring that there is no drug-specific increase in HCC recurrence after HCV cure with DAAs. In a pooled meta-analysis that compared DAAs and IFN-based therapy, the occurrence or recurrence of HCC did not increase after the achievement of SVR (Kutala et al. 2017; Waziry et al. 2017). In one of the largest single studies to date on HCC incidence, 62,354 patients were analyzed retrospectively, and 3271 cases of HCC incidence were identified. In this group, SVR achieved with DAA treatment conveyed a 71% reduction of risk of HCC incidence (Ioannou et al. 2017). Recently, two patients with HCV treated with DAAs with dramatic improvement in HCC tumor burden with antiviral therapy alone were described (Griffith et al. 2018).

### HCC and DAA Treatment Failure

There is some evidence that the presence of active HCC tumors before DAA treatment is associated with low viral response, independent of traditional predictors of HCV treatment failure. In a cohort of 17,487 HCV patients treated with DAAs, there were 482 patients (2.8%) with a history of HCC and 142 liver transplant recipients with pretransplant HCC. Overall SVR was 91.9% in non-HCC, 74.5% in HCC, and 93.4% in liver transplant recipients with HCC. The presence of HCC was associated with a lower likelihood of SVR overall [adjusted odds ratio (AOR) = 0.38] (Beste et al. 2017). In the group of 421 HCV patients with cirrhosis, treatment failure was observed in 29 of 138 (21%) of patients with active or a history of HCC compared to 12% of patients without HCC (p = 0.009). In multivariate analysis, the primary predictor of DAA treatment failure was the presence of active HCC at the time of HCV treatment initiation (AOR = 8.5) (Prenner et al. 2017). The biological explanation for the diminished SVR in patients with active HCC is not entirely clear; HCC cells may serve as a reservoir for HCV in which virus particles may evade DAA therapy.

## Conclusion

Several changes occur in the innate and adaptive immune system during acute and chronic hepatitis C that promote persistence of the infection. Functional polarization of NK cells leading to increased cytolytic activity and decreased IFN-γ synthesis is caused by chronic exposure to IFN-α and may prevent the elimination of the virus and lead to chronic hepatitis. Increased expression of inhibiting receptors, a lack of function of CD4^+^ Th cells, and increased activity of Tregs all contribute to the impaired function of CD8^+^ lymphocytes. Activation of B cells contributes to the development of cryoglobulinemia and non-Hodgkin lymphoma. Inhibition of HCV replication during DAA therapy leads to reversal of NK cells and phenotypic and functional shifts of CD4 and CD8 T cells typical of chronic hepatitis C. These changes during DAA therapy can trigger viral reactivation in patients with chronic hepatitis B and recurrence of HCC in some patients with previous successful cancer treatment.
